# The influence of 10-year Nuss bar placement on bar removal: a case report

**DOI:** 10.1186/s13019-022-02021-3

**Published:** 2022-10-20

**Authors:** Haihua Gu, Guanxin Xu, Tianshu Liu, Sai Zhang

**Affiliations:** Department of Thoracic Surgery, The Second Affiliated Hospital, School of Medicine, Zhejiang University, Jiefang Road 88, Hangzhou, 310009 Zhejiang People’s Republic of China

**Keywords:** Pectus excavatum, Nuss bar removal, Pectus carinatum, Case report

## Abstract

**Background:**

The Nuss bar is commonly used for minimally invasive correction of pectus excavatum and is usually removed within 2–3 years. Here, we report a case of 10-year bar placement after the Nuss procedure accompanied by unique complications of thoracic malformation that have not been described before. The asymmetric pectus carinatum caused by bar displacement and significant rib periosteal hyperplasia is described for the first time.

**Case presentation:**

A 23-year-old man was admitted to our hospital due to the main complaint of obvious chest discomfort when lifting heavy weights. The bar removal was seriously delayed due to his loss to follow-up. Chest asymmetry and distant heart sounds were found during a physical examination. A chest CT scan demonstrated that the right end of the lower bar originally fixed outside the ribs had shifted into the thoracic cavity, and the left costal cartilage was obviously protruding. Additionally, the displaced bars were separated from the sternum and tightly attached to the pericardium, resulting in abnormalities of the anterior mediastinum. These secondary thoracic deformities made the patient extremely prone to massive hemorrhage or multiple rib fractures when sliding the bars out. However, serious consequences were avoided due to reasonable adjustments to the usual bar removal procedures.

**Conclusion:**

This case demonstrates a specific type of bar displacement caused by prolonged placement of the bars and highlights the importance of rigorous follow-up of patients after the Nuss procedure.

## Introduction

The Nuss operation is a minimally invasive and well-established procedure of choice for the correction of pectus excavatum, performed by inserting one or two convex metallic bars beneath the sternum and then turning them over. It is generally accepted that the bars need to be removed 2–3 years after placement [1]. Here, we report a rare case in which the placement time of the pectus bars was prolonged for 10 years due to loss to follow-up. For the first time, asymmetric pectus carinatum caused by bar displacement and significant rib periosteal hyperplasia is described. Therefore, the usual bar removal procedures are not applicable to this case. The outcome of the patient was favorable because of reasonable adjustment of the bar removal procedure to avoid major complications.

## Case presentation

A 23-year-old man had undergone the Nuss procedure with two bars fixation for pectus excavatum at our hospital 10 years ago. The initial plan was to remove the bars 3 years later, but he was lost to follow-up. At present, he was admitted to our hospital due to the main complaint of obvious chest discomfort when lifting heavy weights.

**Fig. 1:**
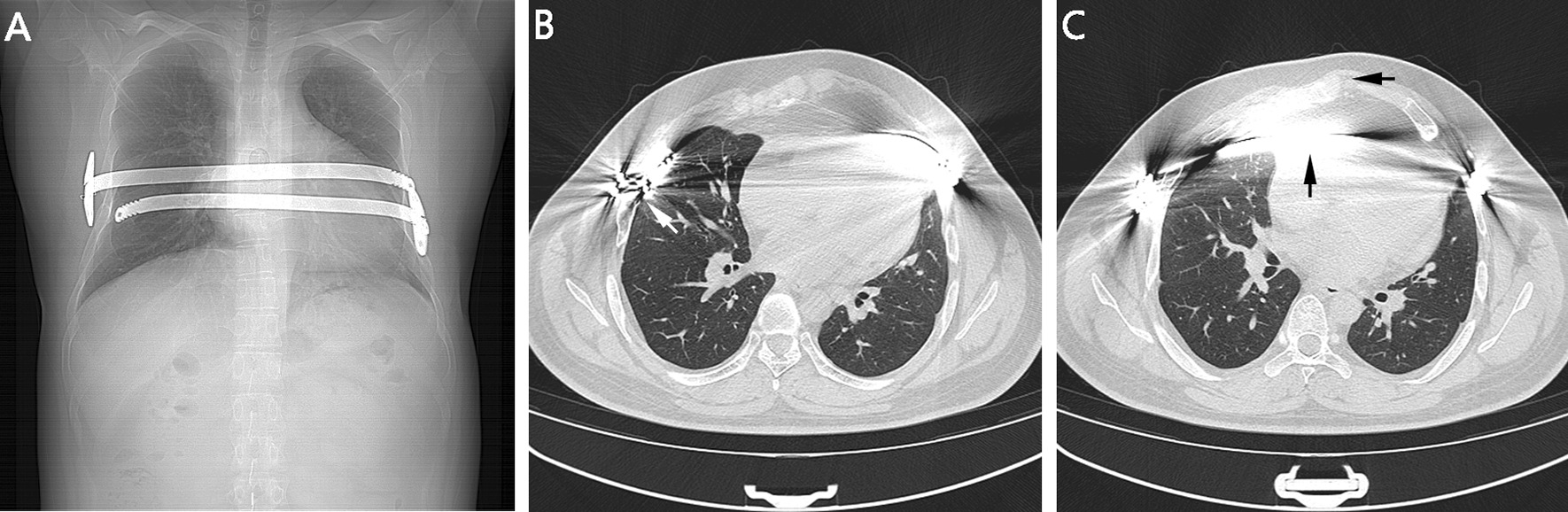
**A** The two bars were obviously too short for the bony thorax. **B** The right end of the lower bar was located inside the ribs (tilted arrow). **C** It shows the abnormalities of the anterior mediastinum. The bars were tightly attached to the pericardium (upward arrow), and the left costal cartilage was obviously protruding (leftward arrow)

Chest asymmetry and distant heart sounds were found during a physical examination. No respiratory symptoms, such as coughing, expectoration, hemoptysis or dyspnea, were observed. A preoperative chest CT scan demonstrated that both sides of the bony thorax were markedly inwardly concave (Fig. 1A). It is also worth noting that the right end of the lower bar originally fixed outside the ribs had shifted into the thoracic cavity, and the left costal cartilage was obviously protruding (Fig. 1B). Additionally, the displaced bars were tightly attached to the pericardium, resulting in abnormalities of the anterior mediastinum (Fig. 1C).

We arranged for the patient to have the bars removed. The patient was positioned in a supine position with a two-sided lateral raise. In the initial surgical scar on both sides, two incisions were made and appropriately extended because of great difficulties in exposing the bars and stabilizers, which were covered and immobilized by large amounts of hyperplastic bone tissue. We scraped off the bone tissue, removed the wires on the stabilizers, and cut off part of the rib for efficient exposure.

**Fig. 2:**
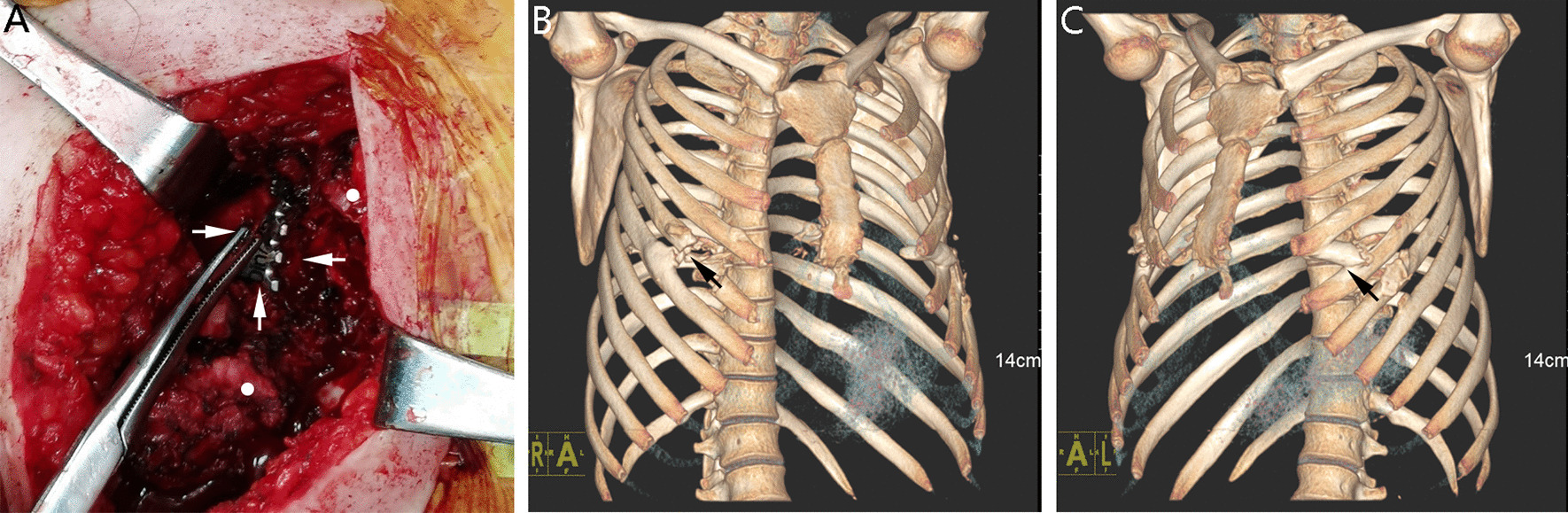
**A** The right end of the lower bar (upward arrow) had shifted into the thoracic cavity (rightward arrow). Large amounts of hyperplastic bone tissue (leftward arrow) was on the inner side of the ribs. Two broken ends (round spot) left after partial excision of the fifth rib. **B** and **C** Three-dimensional reconstruction of rib CT demonstrated the significant rib periosteal hyperplasia from different angles, forming the sheath-like structure around the bars(tilted arrow). The longitudinal axis of the sternum was twisted to the right

It was further clarified that the right end of the lower bar had transferred to the inside of the chest cavity (Fig. 2A). Through the rib defect area, a trocar was inserted, and a 30-degree, 10-mm high definition thoracoscope was used under the ventilation of both lungs. Hyperplastic bone tissue could be seen on the inner side of the local ribs, forming a sheath-like structure around the bars. In addition, we found that the bars had been separated from the sternum and bilateral costal cartilage and were downwardly compressing the pericardium. However, there was no dense adhesion between the bars and the pericardium or lung. The impulse of the heart and the movement of the lungs were unimpeded.

We temporarily withdrew the thoracoscope and gradually removed the hyperplastic bone tissue with a stripper until both ends of the bars were exposed approximately 3 cm in length. Then, a flipper was used to unbend the bars, making them straight enough to remove. The thoracoscope was inserted again, mainly to detect the pericardium and the bilateral bar tracts during the process of rotating the bars slightly. When loosened enough, the bars were slowly withdrawn from the chest. After confirming that there was no pericardial rupture or major bleeding through the bar tracts, we retracted the thoracoscope again, and placed a drainage tube. Local anesthetic was injected, and the incisions were closed in layers.

Pneumothorax was ruled out by chest X-ray taken in the ward. The patient was discharged on the sixth postoperative day. He had a follow-up one month after surgery and reported no chest discomfort. The postoperative rib CT showed that there were no rib fractures (Fig. 2B, C).

## Discussion

The minimally invasive repair of pectus excavatum (MIRPE) is gaining acceptance worldwide and has emerged as the new standard for correcting pectus excavatum [2]. After the introduction of MIRPE, pectus bar dislocation is the most common complication, while other complications, such as pneumothorax, wound infection, seroma, multiple rib fractures, hemothorax, and thoracic outlet-like syndrome are occasionally seen [3]. However, as far as we know, there is only one previous report of serious complications while removing the bars a very long time after the Nuss procedure. Sebastian et al. described a case with life-threatening aortic hemorrhage during the removal of displaced double bars that had been inserted 9 years prior. In that case, rupture of the ascending aorta resulted in profuse bleeding from the left midaxillary incision [4]. Here, we report a rare and impressive complication of thoracic deformity in a man who had undergone MIRPE 10 years prior.

In many cases, pectus carinatum after pectus excavatum repair is probably caused by the pathophysiologic costal cartilage overgrowth and remodeling that underlies the pectus excavatum deformity in affected patients in the first place [5]. Interestingly, the protuberant chest deformity in this case is mainly due to the physical impediment to the development of the chest. With growth during adolescence, the enlarging thorax gradually mismatched the curvature of the bars, resulting in compression of the ribs and stimulating periosteal hyperplasia. The expansion of the thorax led to relative dislocation of the bars and eventually caused the entire bar to move into the chest cavity. In addition, the hyperplastic bone tissue completely wrapped the bars, which limited the development of the ribs and eventually caused local restriction of the chest. With the bars preventing any remodeling trajectory from occurring dorsally, the costal cartilage was projected ventrally [5], ultimately leading to asymmetrical pectus carinatum and a large interspace of the anterior mediastinum, which coincides with the abnormal physical examination. In brief, this is the first case describing asymmetrical pectus carinatum due to the continuing interaction between long-term bar displacement and rib periosteal hyperplasia.

For most patients, Nuss bar removal is a safe, well-tolerated operation that is even suitable as an outpatient procedure [1]. However, a few cases described life-threatening major arterial injury and massive hemorrhage through the bar tract during bar removal [6–8]. Strategies to address these potential complications should be incorporated into presurgical case preparation [7]. First, despite the presence of metal artifacts, chest CT remains an indispensable preoperative workup because it clearly shows the spatial position relationship between the bar and the ribs, which cannot be obtained by X-ray. Because of the rare displacement of the bars and the surrounding heterotopic ossification, this case clearly had the potential for major complications during the process of removing the bars. Although the damage to the internal mammary vessels could be temporarily disregarded because of the large interspace of the anterior mediastinum, the tight proximity of the bars to the pericardium still posed a great risk of aortic hemorrhage or cardiac tamponade.

Second, video-assisted thoracoscopy is also highly recommended for intrathoracic evaluation and observation. In this case, largely due to the abnormal interspace in front of the bars, a 10-mm and 30-degree scope was used in a smooth way without carbon dioxide insufflation or one-lung ventilation.

Third, from the perspective of surgical operations, full exposure of the surgical field, patient removal of hyperplastic bone tissue, and strict control of the speed when pulling out the bars are vital for avoiding serious complications.

Finally, emergency thoracotomy or sternotomy, when necessary, may be difficult and laborious owing to significant rib periosteal hyperplasia and the fixed bars in the chest cavity. In the case of acute pericardial tamponade, a 3 to 4 cm transverse incision should be made immediately below the xiphoid, and a retrosternal tunnel can be created by blunt finger dissection. Due to the interspace of the anterior mediastinum, it would be feasible to perform pericardial fenestration through this tunnel under thoracoscopy.

## Conclusion

Taken together, our case shows that prolonged pectus bar placement caused by loss of follow-up leads to unusual thoracic abnormalities, which makes it extremely difficult to remove the bars. Avoidance of major complications depends largely on the evaluation of preoperative chest CT scans and adjustment of the bar removal procedures through thoracoscopic-assisted exploration. This case highlights the importance of patient education and rigorous follow-up after MIRPE. It should be emphasized that the Nuss procedure is only the first step, and successful bar removal is the real end of the treatment of pectus excavatum.

## Data Availability

The datasets of the current study are available from the corresponding author upon reasonable request.
